# Link between the Nankai underthrust turbidites and shallow slow earthquakes

**DOI:** 10.1038/s41598-023-37474-6

**Published:** 2023-07-10

**Authors:** Jin-Oh Park, Ehsan Jamali Hondori

**Affiliations:** 1 Atmosphere and Ocean Research Institute, University of Tokyo, Kashiwa, Japan; 2 Present Address: Geoscience Enterprise Inc. (GSE), Tokyo, Japan

**Keywords:** Natural hazards, Ocean sciences, Solid Earth sciences

## Abstract

Trench sediments such as pelagic clay or terrigenous turbidites have long been invoked to explain the seismogenic behavior of the megathrust fault (i.e., décollement). Recent numerous studies suggest that slow earthquakes may be associated with huge megathrust earthquake; however, controls on the slow earthquake occurrence remain poorly understood. We investigate seismic reflection data along the Nankai Trough subduction zone to understand the correlations between the spatial distribution of the broad turbidites and along-strike variations in shallow slow earthquakes and slip-deficit rates. This report presents a unique map of regional distribution of the three discrete Miocene turbidites that underthrust apparently along the décollement beneath the Nankai accretionary prism. A comparison of distributions of the Nankai underthrust turbidites, shallow slow earthquakes, and slip-deficit rates enables us to infer that the underthrust turbidites may cause primarily low pore-fluid overpressures and high effective vertical stresses across the décollement, leading to potentially inhibiting the slow earthquake occurrence. Our findings provide a new insight into potential role of the underthrust turbidites for shallow slow earthquakes at subduction zone.

## Introduction

**Figure 1:**
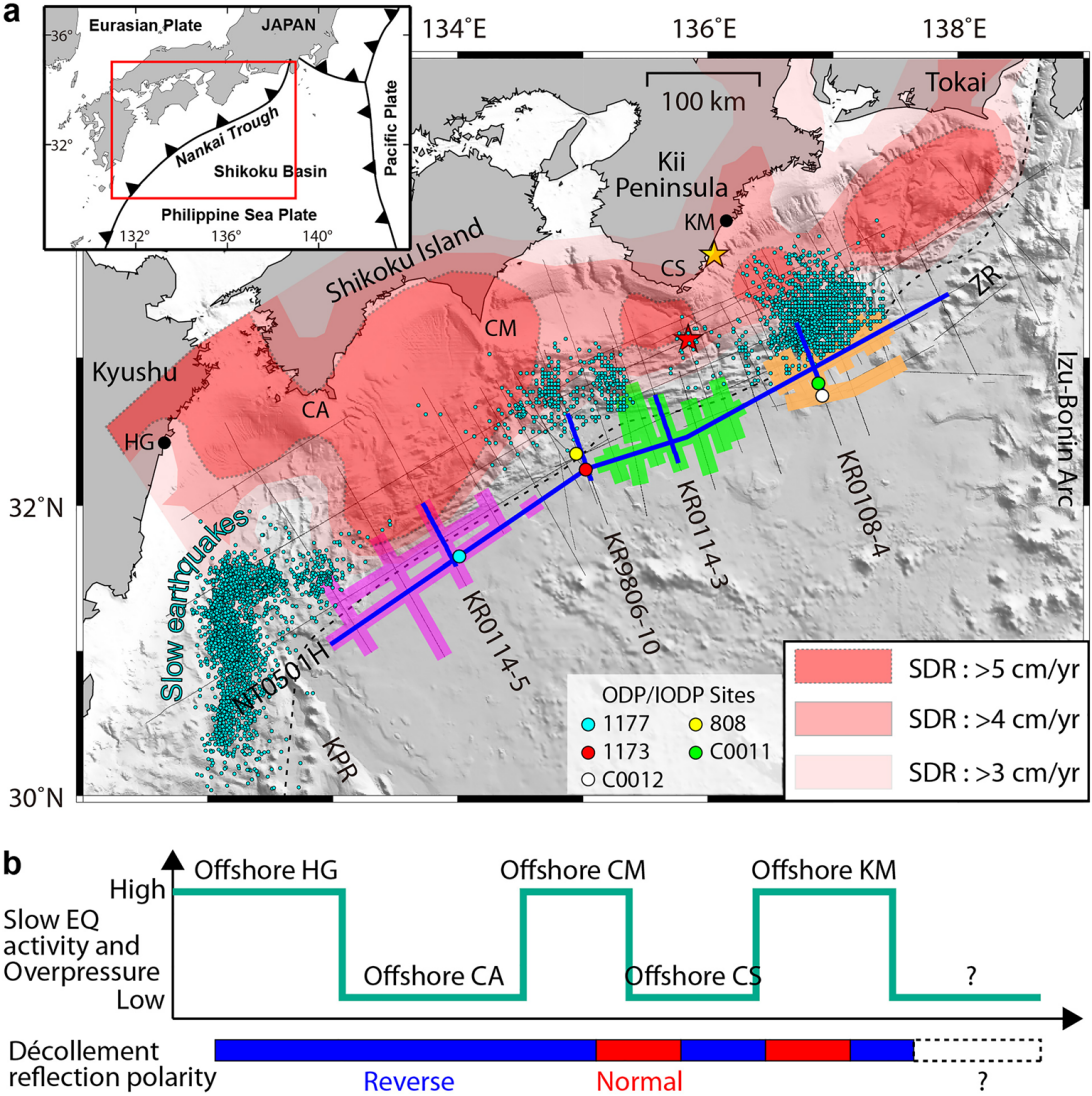
Seafloor topography, physiographic features, MCS lines, turbidite distribution, slow earthquake activity, and décollement properties in the Nankai Trough margin offshore southwest Japan. (**a**) Inset: Regional map showing the location of the study area (red box). Gray thin lines mark the locations of the MCS survey lines. Blue thick parts on MCS lines mark the MCS profiles shown in Figs. 2 and 3. Thick purple, green, and orange parts on dip and strike MCS lines mark western turbidite (WT), central turbidite (CT), and eastern turbidite (ET) facies within the Shikoku Basin sedimentary section, respectively. Slip-deficit rate (SDR) distributions^1^ are shaded in red. Dark cyan dots mark shallow slow earthquakes of very low frequency earthquakes^5–8^ and low-frequency tremors^9, 10^. A black dotted line marks the trench axis (i.e., frontal thrust). Yellow and red stars show epicenters of the 1944 Tonankai (M8.1) and 1946 Nankai (M8.3) earthquakes, respectively. HG, Hyuga; CA, Cape Ashizuri; CM, Cape Muroto; CS, Cape Shiono; KM, Kumano; KPR, Kyushu-Palau ridge; ZR, Zenisu ridge. (**b**) (Upper) Schematic graph of slow earthquake (EQ) activity and pore-fluid overpressure levels and (Lower) décollement reflection polarities^11^ along the Nankai shallow subduction zone.

Megathrust earthquakes are the result of relative plate motion and occur at slip-deficit (i.e., plate coupling) regions^1, 2^, where friction prevents plates from slipping against each other and the accumulated elastic strain is eventually released. Recent seafloor geodetic observations have revealed variation in interplate coupling along the Nankai subduction zone^1^. Slow earthquakes may be associated with huge megathrust earthquake generation in that they can have common slip mechanisms and are located in neighboring regions of the seismogenic zone^2^. The Nankai Trough subduction zone (Fig. 1a) offshore southwest Japan has been known as one of the best convergent margins to study slow earthquakes as well as large megathrust earthquakes (for example, the 1944 Tonankai (M8.1) and 1946 Nankai (M8.3) events) with a recurrence interval of 100–200 years^3^. In fact, Japanese government is concerned about strong motion and tsunamis owing to a future M8 to M9 megathrust earthquake along the Nankai Trough in a probability of ~ 90% within the next 40 years^4^.

Several kinds of slow earthquakes have occurred along the Nankai subduction zone^2^. Particularly, the Nankai shallow subduction zone (Fig. 1a) is characterized by very low frequency earthquakes (VLFEs)^5–8^ and low frequency tremors^9, 10^. The Nankai shallow slow earthquakes appear to be almost localized around the high slip-deficit rate (SDR) region (Fig. 1a). Previous studies have tried to explain the clustered distribution of the Nankai shallow slow earthquakes by subduction of basement highs such as ridge or seamounts that might be a structural factor controlling the excitation conditions of slow earthquakes; for example, many slow events around the subducting Kyushu-Palau ridge offshore Hyuga of the Kyushu in the western Nankai^1, 10^. However, those subducting structural features alone are not likely able to sufficiently explain slow earthquake activities; for example, few slow earthquakes have occurred around the subducted Paleo-Zenisu ridge offshore the Tokai region in the eastern Nankai^1, 12^. It has been hypothesized that high pore-fluid pressures within forearc accretionary wedge, which are inferred from low seismic velocities, may cause the shallow slow earthquakes^6, 8, 13^. However, there is a lack of comprehensive understanding on the nature of pore-fluid pressures associated with the megathrust fault (i.e., décollement) slip to generate slow earthquakes. For example, few studies have quantified pore-fluid pressures necessary for exciting slow earthquakes so far. Moreover, low activity of shallow slow earthquakes offshore Cape Ashizuri (CA) of the Shikoku Island cannot be simply explained by probably elevated pore-fluid pressures, which are suggested by reversed-polarity reflection of the décollement (Fig. 1b). Therefore, controls on the clustered distribution of the Nankai shallow slow earthquakes are still poorly understood.

At the Nankai Trough margin (Fig. 1a), the Philippine Sea Plate (PSP) is being subducted beneath the Eurasian Plate to the northwest at a convergence rate of ~ 4 cm/year (Ref.^14^). The Shikoku Basin, the northern part of the PSP, is estimated to have opened between 25 and 15 Ma by backarc spreading of the Izu-Bonin arc^15^. The > 100 km wide Nankai accretionary prism, which has developed landward of the trench since the Miocene, mainly consists of offscraped and underplated materials from the trough-fill turbidites and the Shikoku Basin sediments^16^. Incoming sediments to subduction zones get involved in not only the growth and recycling of continental crust but also megathrust and/or slow earthquake occurrence through the accretion process^17–23^. Trench sediments such as pelagic clay or terrigenous turbidite have long been invoked to explain the seismogenic behavior of the megathrust fault, for example, the Middle America Trench^24^, the Sunda Trench^25^, and the Japan Trench^26^. A numerical modeling study suggested that lithostratigraphy of the underthrust sediment can control dewatering and pore-fluid pressure in the footwall of the western Nankai, where thick turbidite sequence subducts^27^. Although many geological and geophysical investigations^11, 28–31^ have identified the turbidites, few studies have obtained a complete view of the turbidite distribution for the entire Nankai subduction zone, except for the Tilley et al.^32^ where the turbidite distribution is largely confined to regions seaward of the trench axis. Moreover, a possible link between the underthrust turbidites and shallow slow earthquakes remains less well documented for the entire Nankai subduction zone.

In this paper we show several multi-channel seismic (MCS) reflection profiles that reveal incoming turbidites facies to the Nankai subduction zone, and document along-strike variations in the underthrust sediments, décollement properties, and slow earthquakes. Moreover, we present potential implications of the underthrust turbidites for the shallow slow earthquakes and fault strength of the décollement.

## Results

We performed lithostratigraphic interpretation on the MCS profiles (Fig. 1a, “Methods” section) in the Nankai Trough margin. Based on seismic reflection characteristics and Ocean Drilling Program (ODP)/Integrated Ocean Drilling Program (IODP) drilling results^29, 33^, we identify four specific seismic reflection units within the Shikoku Basin sedimentary section (i.e., hemipelagic mudstone) on the oceanic crust: units A, B, C, and D from west to east.

### Turbidites on a seismic profile along the Nankai Trough

**Figure 2:**
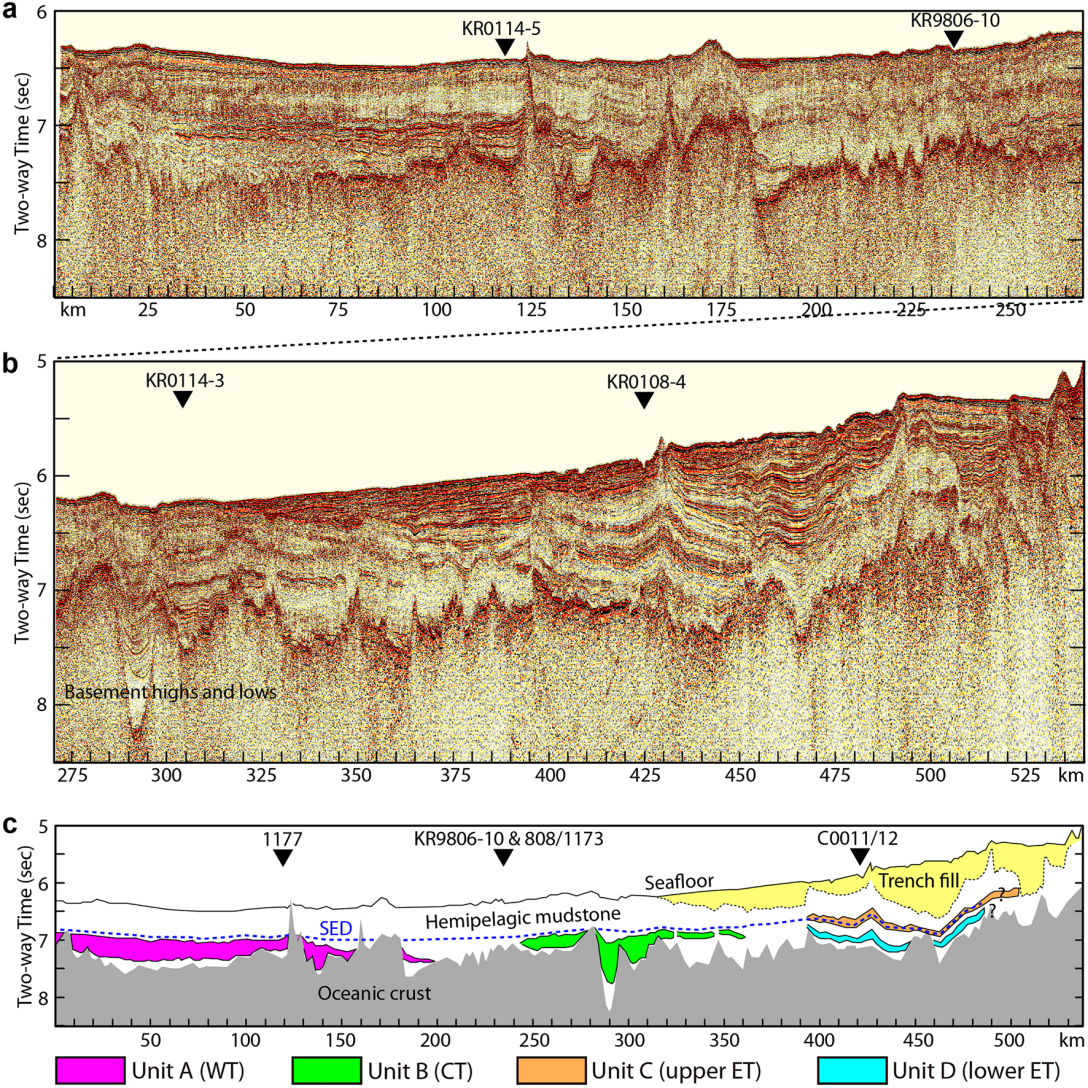
Time-migrated MCS profile of line NT0501H and interpretation. (**a**) Western half of the MCS line NT0501H perpendicular to MCS lines KR0114-5 and KR9806-10. Vertical exaggeration about 40:1 at the seafloor. (**b**) Eastern half of the MCS line NT0501H perpendicular to MCS lines KR0114-3 and KR0108-4. (**c**) Interpretation of the entire MCS line NT0501H showing four major seismic units above oceanic crust: unit A (WT, western turbidite), unit B (CT, central turbidite), unit C (upper ET, upper eastern turbidite), and unit D (lower ET, lower eastern turbidite). The Stratigraphic equivalent of the décollement (SED) is shown in blue dotted line. Three ODP sites (1177, 808, and 1173) and two IODP sites (C0011 and C0012) are projected.

On the ~ 540-km-long MCS profile of line NT0501H (Fig. 2) along the Nankai Trough, we can recognize ~ 200-km-wide unit A with middle to late Miocene age offshore Cape Ashizuri (CA) of the Shikoku Island. Sheet-like unit A exhibits alterations of high and low amplitudes, and continuous reflections. Drilling at ODP Site 1177 recovered ~ 299-m-thick siliciclastic turbidite packages consisting of mudstones and sandstones for this unit A. The mudstone beds, sandwiched both above and below by more permeable sandstones, are inferred to undergo dewatering in the underthrust sediment, because laterally continuous sandstones may act as effective drainages for the dewatering. Pickering et al.^31^ named this unit Kyushu Fan. Hereinafter, we call the unit A as ‘western turbidite’ (WT).

We observe ~ 120-km-wide unit B offshore Cape Shiono (CS) of the Kii Peninsula on the MCS profile of line NT0501H. Unit B is composed of four discrete channel bodies that are lens-shaped or filling basement lows. This unit shows relatively continuous and partially chaotic reflections. Underwood and Pickering^34^ interpreted unit B to be equivalent to Kyushu Fan (turbidites), even though its geologic age and lithology are unknown due to lack of drilling through this unit. In order to distinguish it from unit A, we call the unit B as ‘central turbidite’ (CT).

We identify two different units C and D offshore Kumano of the east Kii Peninsula, which are ~ 100 km wide. Units C and D are characterized by discontinuous reflections that have a higher amplitude than the surrounding strata. Drilling at IODP Site C0011 recovered ~ 140-m-thick volcanic turbidite with late Miocene age for unit C (Zenisu Fan; upper eastern turbidite), ~ 195-m-thick hemipelagic mudstone, and ~ 176-m-thick mixed (siliciclastic and volcaniclastic) turbidite with middle Miocene age for unit D (Kyushu Fan; lower eastern turbidite) from top to bottom^31^. In fact, the Kyushu Fan at Site C0011 may be thicker than the ~ 176 m, because the drilling stopped within the turbidite section so that the sediment-basement interface was not cored. Hereinafter, we call an entire layer from units C down to D as ‘eastern turbidite’ (ET), which includes the interbedded hemipelagic mudstone facies.

### Underthrust turbidites on seismic profiles across the Nankai Trough

**Figure 3:**
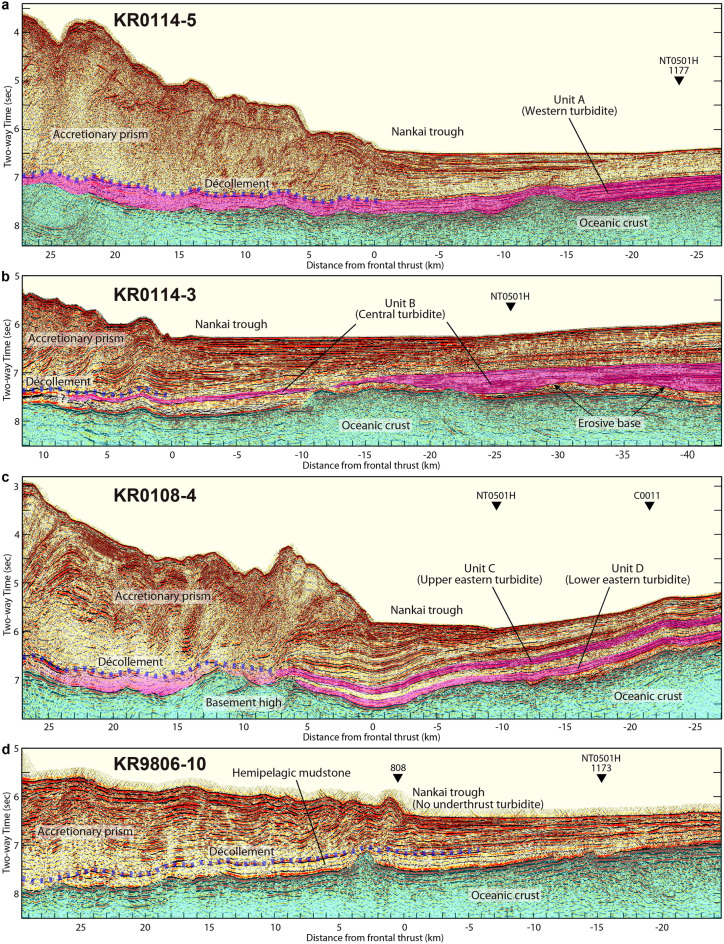
Time-migrated MCS profiles with interpretations perpendicular to the MCS line NT0501H. Vertical exaggeration about 5:1 at the seafloor. The Subducting oceanic crust of the PSP is shaded in light cyan. (**a**) MCS profile of line KR0114-5. Unit A (western turbidite) subducting beneath the décollement (blue shaded dots) is shaded in pink. Note reverse-polarity reflection of the décollement (red-black-red), compared with seafloor (black-red-black). An ODP site (1177) is projected. (**b**) MCS profile of line KR0114-3. Unit B (central turbidite) subducting beneath the décollement (blue shaded dots) is shaded in pink. We observe erosive bases at the bottom of unit B. Note normal-polarity reflection of the décollement (black-red-black), compared with seafloor (black-red-black). (**c**) MCS profile of line KR0108-4. Units C (upper eastern turbidite) and D (lower eastern turbidite) subducting beneath the décollement (blue shaded dots) are shaded in pink. Note normal-polarity reflection of the décollement (black-red-black), compared with seafloor (black-red-black). An IODP Site C0011 is projected. (**d**) MCS profile of line KR9806-10. Hemipelagic mudstone underthrusts beneath the décollement (blue shaded dots). Note reverse-polarity reflection of the décollement (red-black-red), compared with seafloor (black-red-black). Two ODP sites (808 and 1173) are projected.

On the MCS profiles crossing the line NT0501H and trench axis, we identify the same units A, B, C, and D (namely WT, CT, and ET) subducting along the décollement beneath the Nankai Trough accretionary prism. We observe the sheet-like unit A (WT) subducting beneath the accretionary prism on the MCS profile of line KR0114-5 (Fig. 3a, Fig. S1a in supplementary material) offshore CA. The décollement appears to occur on or above the upper boundary of the WT, indicating a complete underthrust of the WT. On the MCS profile of line KR0114-3 (Fig. 3b, Fig. S1b in supplementary material) offshore CS, we observe the lens-shaped or channel-filling unit B (CT) with erosive bases, which subducts beneath the accretionary prism. The décollement appears to occur on or above the CT, indicating fully underthrusting CT. On the MCS profile of line KR0108-4 (Fig. 3c, Fig. S1c in supplementary material) offshore Kumano, we identify the units C and D interbedded with the hemipelagic mudstone facies as recovered at Site C0011, which subduct beneath the accretionary prism. The décollement appears to occur in unit C (upper ET), indicating partial underthrust of unit C and complete underthrust of the hemipelagic mudstone layer and unit D (lower ET). In contrast, no turbidite unit subducts beneath the accretionary prism on the MCS profile of line KR9806-10 (Fig. 3d, Fig. S1d in supplementary material) offshore Cape Muroto (CM) of the Shikoku Island. Drillings at ODP Sites 808 and 1173 offshore CM recovered monotonous mudstones with low permeability beneath the décollement.

In summary, Fig. 1a presents a unique map of regional distribution of the three discrete turbidites (WT, CT, and ET) for the entire Nankai Trough margin, which is compiled by the MCS and drilling data. We observe the underthrust turbidites over ~ 420 km (ca. 78%) on a ~ 540-km-long profile east–west along the Nankai Trough.

## Discussion

Based on seismic reflection characteristics and ocean drilling results, we mapped the three discrete turbidites (WT, CT, and ET) with Miocene age for the entire Nankai subduction zone. As earlier studies on the Nankai Trough turbidites, Ike et al.^30^ documented regional and local variations in basement relief, sediment thickness, and sediment type in the Shikoku Basin. They focused on turbidite sedimentation in the Shikoku Basin on incoming Philippine Sea Plate, which reflects basement control on deposition, leading to the local presence or absence of turbidite units. Tilley et al.^32^ investigated the seismic reflection characteristics of incoming sediments to know sediment and basement variations related to the along-strike changes within the accretionary prism and plate boundary conditions at the Nankai Trough. In this paper we spatially mapped the turbidites and expanded their distribution landward from the Nankai Trough to investigate their control on seismogenic behavior of the décollement with integration of the turbidite subduction, slip-deficit rates, slow earthquakes, the décollement reflection polarities, and surface slope angles of the accretionary wedge.

### Potential role of the underthrust turbidites for slow earthquakes

Comparing spatial distribution of the underthrusting turbidites to slow earthquakes (Fig. 1a), we found that forearc regions offshore CA and CS, where the underthrusting WT and CT are expected to extend landward respectively, are largely consistent with the low activity of slow earthquakes (Fig. 1b). These regions are almost characterized by high SDRs indicative of high interplate coupling conditions, which were determined by seafloor geodetic observations^1^. In contrast, many slow earthquakes have been observed in a forearc region offshore CM which monotonous mudstones with low permeability subduct beneath the accretionary prism, without the underthrusting turbidites. A numerical simulation study^27^ on dewatering and pore-fluid pressure of subducting sedimentary sequence demonstrated that sandstone-dominated system with enhanced drainage has lower pore-fluid overpressures and higher effective stresses than mudstone-dominated system, leading to lower porosity and greater stiffness. The numerical study also suggests that the less-permeable mudstone layer could be poorly drained and likely to be much overpressured, leading to low effective stresses in the footwall beneath the décollement. Assuming that the shallow slow earthquakes are closely associated with high pore-fluid pressure^8^ as well as material heterogeneity, geometric complexity and deformation at low differential stress^35^, we infer that the Nankai underthrust turbidites containing permeable sandstones may cause primarily low pore-fluid overpressures and high effective vertical stresses across the décollement, leading to high interplate coupling and thus potentially inhibiting the shallow slow earthquakes. This inference is consistent with simulated pore-fluid pressures^36^ that result in basal shear strengths reaching ~ 20 MPa at 30 km landward from the trench offshore CA and only ~ 5 MPa at the same distance offshore CM. Elevated pore pressures reduce the effective vertical stress and thus lower the shear stress necessary for failure of the décollement.

### Along-strike variations in the underthrust sediments, décollement properties, and slow earthquakes

Reflection polarities (reverse or normal) of the décollement suggest states of physical properties along the décollement and beneath it^11^, which may influence pore-fluid pressures of the décollement and therefore slow earthquakes. The reverse polarity reflection (e.g., Fig. 3a,d) is caused by a decrease in acoustic impedance (velocity × density) across the décollement as an interface between a high-impedance hanging wall (accretionary prism) and a low-impedance footwall (underthrust sediment). The reverse-polarity reflection simply suggests that the décollement and underthrust sediment are poorly drained and highly overpressured. The normal polarity reflection (e.g., Fig. 3b,c) is caused by an increase in acoustic impedance across the décollement between a low-impedance hanging wall and a high-impedance footwall. A previous study by Park et al.^11^ revealed a variation in the décollement reflection polarities along the Nankai subduction zone; however, controls on the variation remained unclear. Here we propose that the décollement reflection polarities are more likely related to variations in the turbidite subduction, pore-fluid overpressures, and slow earthquakes along the Nankai shallow subduction zone, as below.

Offshore Hyuga of the Kyushu and CM of the Shikoku Island, many shallow slow earthquakes have been observed (Fig. 1a). It is likely related to high pore-fluid overpressures and thus reverse-polarity reflection (Fig. 1b) of the décollement, consistent with lack of underthrust turbidites. Offshore CA of the Shikoku Island, we unexpectedly observed the reverse-polarity reflection of the décollement, despite a correlation of three phenomena (underthrust turbidites, potentially low pore-fluid overpressures, and few slow earthquakes). A possible scenario to explain this is that the décollement offshore CA might be somewhat overpressured despite the underthrusting WT so that it can exhibit the reverse-polarity reflection, but its overpressure is not actually that high enough to facilitate the slow earthquake activity.

Offshore CS of the Kii Peninsula, we observed alterations of normal and reverse polarity reflections of the décollement. Consistent with low activity of the shallow slow earthquake, the normal-polarity reflection inherently suggestive of low pore-fluid overpressure may be attributed to the underthrust CT. In contrast, the reverse-polarity reflection could be caused by the underthrust CT deposited within isolated basement lows and erosional channels (Figs. 2b and 3b) that may generate local compartments of overpressure in the footwall, because the turbidites pinch out against the flanks of surrounding topographic highs and thus their pore-fluid escape could be inhibited^32, 37, 38^. Nevertheless, the overpressure appears not to be that high enough to cause many slow earthquakes.

Control of the underthrust turbidites on the décollement reflection polarity and shallow slow earthquakes appears more complicated offshore Kumano of the east Kii Peninsula where we observe alterations of normal and reverse polarity reflections of the décollement. Because the décollement occurs within the underthrust upper ET (i.e., unit C on MCS line KR0108-4, see Fig. 3c) with possibly enhanced drainage, we can simply expect low pore-fluid overpressures and high effective stresses developing in the footwall, leading to low activity of slow earthquakes and the normal-polarity reflection of the décollement. As a matter of fact, however, many shallow slow earthquakes have been observed offshore Kumano. Moreover, reflections of reverse polarity as well as normal polarity are observed along the décollement. A possible explanation for this unexpected case is that the underthrust ET containing the massive less-permeable mudstone layer (e.g., ~ 195 m thick at Site C0011) may cause relatively high pore pressures and low effective stresses across the décollement, favoring the reverse-polarity reflection and slow earthquakes. Moreover, the channel deposits^31^ of the upper ET would help to maintain the overpressured décollement. Alternatively, such frequent shallow slow earthquakes offshore Kumano may be associated with: (1) a subducting ridge^12^ potentially creating a complicated fracture network and thus generating slow earthquakes; (2) the presence of a low seismic velocity zone^16^ suggesting high fluid pressures^39^ and thus favoring slow earthquakes; (3) two large intra-slab earthquakes (M7.2 and 7.5) with thrust-type focal mechanism that occurred offshore Kumano in 2004, which may have induced the pore-fluid migration along the pre-existing faults or created a new fracture network, thereby triggering the slow earthquakes^5^.

### Décollement fault strength estimation by a simple topographic parameter

**Figure 4:**
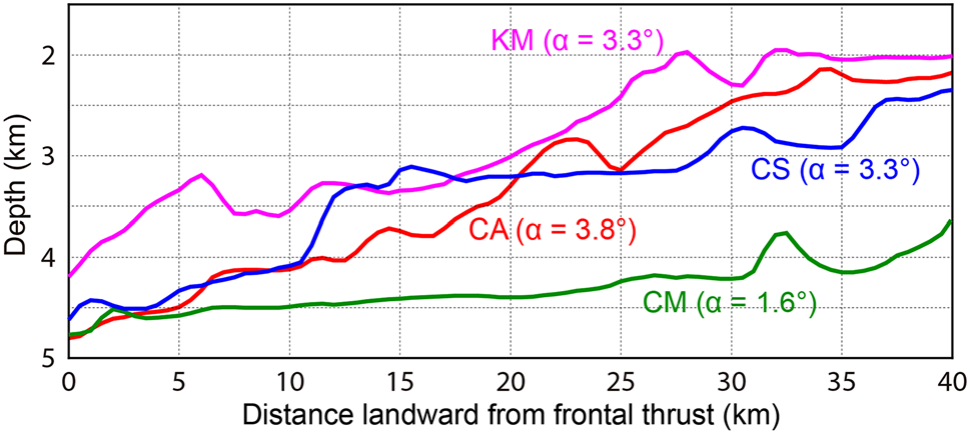
Representative bathymetry profiles and seafloor surface slope angles (α) for accretionary wedges offshore Cape Ashizuri (CA), Cape Shiono (CS), Cape Muroto (CM), and Kumano (KM). Corresponding MCS lines are KR0114-5 offshore CA, KR0114-3 offshore CS, KR9806-10 offshore CM, and KR0108-4 offshore KM. The surface slope angle (α) is averaged for 40-km-long accretionary wedge profile on each MCS line.

Wedge taper angles (surface slope α plus basal dip β) are largely controlled by the frictional strength of the décollement^40^. A recent theoretical study on critical taper model demonstrated that seafloor surface slope angle could be a first-order approximation to account for effective coefficient of basal friction for convergent margin wedge and thus the strength of décollement when the pore-fluid pressure ratio (λ) is high, internal friction (φ) is small, or both^41^. Koge et al.^41^ calculated WOA (weight of alpha (α)) of 70% based on (λ, φ) = (0.7, 27°), and therefore suggested that their method can be applicable to the Nankai outer accretionary wedge. We accepted their suggestion because the Nankai internal friction (φ = 27°) is much small although the pore-fluid pressure ratio (λ = 0.7) is not that high, compared to other 21 subduction zones with average values (λ, φ) = (0.88, 34°) for which the theoretical study was done. In fact, the strength or pore-fluid pressure conditions in the footwall of the décollement do not directly govern the critical taper model, because the frictional strength of the décollement is affected by frictional coefficient which is a product of intrinsic (or material) friction coefficient and pore-fluid pressure. To simplify the argument over the variation in basal friction strength across the Nankai margin, we assume that physical properties in the footwall primarily affect frictional strength of the décollement. This assumption is consistent with the numerical study^27^ suggesting that sediments in the underthrust sequence may hold implications for the overlying décollement, as they mediate the mechanical strength and elastic strain accumulation in the footwall. Average surface slope angles (α = 3.3 to 3.8°) of the Nankai outer ~ 40-km-wide accretionary wedge offshore CA and CS, characterized by subduction of the WT and CT respectively, are higher than that offshore CM (α = 1.6°) (Fig. 4), suggesting the stronger shear strength of décollement offshore CA and CS. This supports our inference that the underthrust turbidites and expected low pore-fluid overpressure and high effective stress across the décollement probably play an important role in facilitating the interplate coupling and eventually reducing shallow slow earthquakes offshore CA and CS. In contrast, the décollements offshore Hyuga, CM, and Kumano are weakly coupled and prone to slip. Interestingly, the average surface slope angle (α = 3.3°) offshore Kumano is relatively high and very similar to that offshore CS (Fig. 4), even though the surface angle is expected to be lower because of potentially overpressured décollement. We speculate that both a subduction of basement high below the frontal prism (~ 5 to 15 km distance in Fig. 3c) and steep splay fault in the outer ridge^42^ might help growth of the surface slope angle.

Tilley et al.^32^ argued that the wedge taper may be more correlative (correlation coefficient ρ = 0.61) with the thickness of the mud-dominant facies than the turbidite thickness, referring to much low correlation (ρ = 0.17) between the turbidite thickness and wedge taper angle. When we focus on the underthrust sediment facies (permeable sand-dominant (i.e., turbidite) or less permeable mud-dominant) potentially affecting the wedge taper, their results are in contrast with not only our inference above but also previous studies^27, 29, 36^ that the mud-dominant sedimentary layer would maintain high pore pressures and low wedge tapers. The low correlation (ρ = 0.17) between the turbidite thickness and wedge taper angle could be underestimated probably because their interpretation on the underthrust turbidites offshore CS and Kumano was almost limited to the incoming sequence to the Nankai Trough, which is much improved in our study. A recent preliminary estimation of pore-fluid pressures using seismic interval velocities (i.e., velocity–porosity–effective stress transformations) along the Nankai décollement^43^ showed a general trend of higher pore pressures for the décollement of mudstone-dominated system offshore CM than that of sandstone-dominated system offshore CS, still supporting our inference above and the previous studies.

### Summary and future perspectives

We investigate seismic reflection data along the Nankai Trough subduction zone to understand the correlations between the spatial distribution of the three broad underthrust turbidites and along-strike variations in shallow slow earthquakes (very low frequency earthquakes (VLFEs) and low frequency tremors) and slip-deficit rates. We find that while the first two turbidites seem to correlate with a lack of VLFE activity and high slip-deficit rates along strike, the third easternmost turbidite is more complicated and correlate with the VLFE occurrence. We speculate that the underthrust turbidites with potentially permeable sandstones may cause low pore-fluid overpressures and high effective vertical stresses across the décollement, leading to inhibiting the slow earthquake occurrence. However, the connection between the underthrust turbidite and the fault slip mechanics that may produce VLFEs along the décollement is not established yet. Moreover, pore-fluid pressure of the décollement is not the only factor that can lead to slow earthquakes, although high pore-fluid pressure has been hypothesized to be a key factor in slow earthquake occurrence at subduction zones. As a future study, quantitative and seamless comparison of the in-situ pore pressures over the broader Nankai décollement would be required to validate our inference. Laboratory experiments to test the frictional behavior of the Nankai décollement samples recovered in the sandstone-dominated (i.e., turbidite) and mudstone-dominated systems would be useful for understanding on fault slip behavior associated with the shallow slow earthquakes.

## Methods

### Seismic reflection data acquisition and processing

The MCS data presented in this paper (Fig. 1a) were acquired by R/V *Kairei* of the Japan Marine Science and Technology Center (JAMSTEC) from 1997 through 2010. For deep-penetration seismic imaging, a large volume (~ 200 L) air gun array was used as the controlled sound source, except for a trench-parallel line of NT0501H on which two GI guns (~ 12 L) were used to obtain high resolution images of the incoming sedimentary layer to the Nankai Trough. The MCS data were recorded with a 4000 m, 160-channel streamer with 25 m group spacing, except for the line of NT0501H with a 5100 m, 204-channel streamer. Conventional MCS data processing techniques were applied to all the data, including trace editing, pre-filtering, spherical divergence correction, signature deconvolution, common midpoint (CMP) sort, normal moveout correction, multiple suppression by parabolic radon transform, CMP stack, and Kirchhoff post-stack time migration.

## Supplementary Information

Supplementary Figure S1.

## Data Availability

The seismic reflection data used in this research can be requested from the JAMSTEC Seismic Survey Database (https://www.jamstec.go.jp/obsmcs_db/e/). The Nankai shallow slow earthquake data from previous works are obtained from the Slow Earthquake Database (Kano et al., 2018; http://www-solid.eps.s.u-tokyo.ac.jp/~sloweq/).

## References

[1] Yokota Y, Ishikawa T, Watanabe S, Tashiro T, Asada A (2016). Seafloor geodetic constraints on interplate coupling of the Nankai Trough megathrust zone. Nature.

[2] Obara K, Kato A (2016). Connecting slow earthquakes to huge earthquakes. Science.

[3] Ando M (1975). Source mechanisms and tectonic significance of historical earthquakes along the Nankai Trough, Japan. Tectonophysics.

[4] Japanese government’s Earthquake Research Committee. Updates on earthquake potential by long-term evaluation in and around Japanese Islands. [Available at https://www.static.jishin.go.jp/resource/evaluation/long_term_evaluation/updates/prob2022.pdf] (2022).

[5] Obara K, Ito Y (2005). Very low frequency earthquakes excited by the 2004 off the Kii peninsula earthquakes: A dynamic deformation process in the large accretionary prism. Earth Planets Space.

[6] Ito Y, Obara K (2006). Dynamic deformation of the accretionary prism excites very low frequency earthquakes. Geophys. Res. Lett..

[7] Nakano M, Hori T, Araki E, Kodaira S, Ide S (2018). Shallow very-low frequency earthquakes accompany slow slip events in the Nankai subduction zone. Nat. Commun..

[8] Takemura S (2019). Migrations and clusters of shallow very low frequency earthquakes in the regions surrounding shear stress accumulation peaks along the Nankai Trough. Geophys. Res. Lett..

[9] Yamashita Y (2015). Migrating tremor off southern Kyushu as evidence for slow slip of a shallow subduction interface. Science.

[10] Tonegawa T (2020). Spatial relationship between shallow very low frequency earthquakes and the subducted Kyushu-Palau Ridge in the Hyuga-nada region of the Nankai subduction zone. Geophys. J. Int..

[11] Park J-O, Naruse H, Bangs NL (2014). Along-strike variations in the Nankai shallow décollement properties and their implications for tsunami earthquake generation. Geophys. Res. Lett..

[12] Park J-O, Moore GF, Tsuru T, Kodaira S, Kaneda Y (2004). A subducted oceanic ridge influencing the Nankai megathrust earthquake rupture. Earth Planet. Sci. Lett..

[13] Tonegawa T (2017). Sporadic low-velocity volumes spatially correlate with shallow very low frequency earthquake clusters. Nat. Commun..

[14] Seno T, Stein S, Gripp AE (1993). A model for the motion of the Philippine Sea plate consistent with NUVEL-1 and geological data. J. Geophys. Res..

[15] Okino K, Shimakawa Y, Nagaoka S (1994). Evolution of the Shikoku basin. J. Geomagn. Geoelectr..

[16] Park J-O (2010). A low-velocity zone with weak reflectivity along the Nankai subduction zone. Geology.

[17] Moore JC, Silver EA (1987). Continental margin tectonics: Submarine accretionary prisms. Rev. Geophys..

[18] Ruff L (1989). Do trench sediments affect great earthquake occurrence in subduction zones?. Pure Appl. Geophys..

[19] Lay T (2012). Depth-varying rupture properties of subduction zone megathrust faults. J. Geophys. Res..

[20] Heuret A, Conrad CP, Funiciello F, Lallemand S, Sandri L (2012). Relation between subduction megathrust earthquakes, trench sediment thickness and upper plate strain. Geophys. Res. Lett..

[21] Liu X, Zhao D (2018). Upper and lower plate controls on the great 2011 Tohoku-Oki earthquake. Sci. Adv..

[22] Brizzi S, Zelst I, Funiciello F, Corbi F, Dinther Y (2020). How sediment thickness influences subduction dynamics and seismicity. J. Geophys. Res..

[23] Nakata R, Hori T, Miura S, Hino R (2021). Presence of interplate channel layer controls of slip during and after the 2011 Tohoku-Oki earthquake through the frictional characteristics. Sci. Rep..

[24] Kanamori H, Kikuchi M (1993). The 1992 Nicaragua earthquake: A slow tsunami earthquake associated with subducted sediments. Nature.

[25] Dean SM (2010). Contrasting décollement development and prism deformation across the Sumatra 2004–2005 earthquake rupture boundary. Science.

[26] Chester FM (2013). Structure and composition of the plate-boundary slip zone for the 2011 Tohoku-Oki earthquake. Science.

[27] Hüpers A, Saffer DM, Kopf AJ (2018). Lithostratigraphic controls on dewatering and fluid pressure in the western Nankai subduction zone: Implications for the drainage behavior and consolidation state of the underthrust sequence. Geol. Soc. Am. Spec. Pap..

[28] Moore GF (1990). Structure of the Nankai Trough accretionary zone from multichannel seismic reflection data. J. Geophys. Res..

[29] Moore GF (2001). New insights into deformation and fluid flow processes in the Nankai Trough accretionary prism: Results of ocean drilling program leg 190. Geochem. Geophys. Geosyst..

[30] Ike T (2008). Variations in sediment thickness and type along the northern Philippine Sea plate at the Nankai Trough. Island Arc.

[31] Pickering KT (2013). Depositional architecture, provenance, and tectonic/eustatic modulation of Miocene submarine fans in the Shikoku Basin: Results from the Nankai Trough seismogenic zone experiment. Geochem. Geophys. Geosyst..

[32] Tilley H (2021). Heterogeneous sediment input at the Nankai Trough subduction zone: Implications for shallow slow earthquake localization. Geochem. Geophys. Geosyst..

[33] Saito, S., Underwood, M. B., Kubo, Y. & the Expedition 322 Scientists. NanTroSEIZE stage 2: Subduction inputs. In *Proc. IODP 322*, Tokyo (Integrated Ocean Drilling Program Management International, Inc.) (2010).

[34] Underwood MB, Pickering KT (2018). Facies architecture, detrital provenance, and tectonic modulation of sedimentation in the Shikoku Basin: Inputs to the Nankai Trough subduction zone. Geol. Soc. Am. Spec. Pap..

[35] Kirkpatrick JD, Fagereng Å, Shelly DR (2021). Geological constraints on the mechanisms of slow earthquakes. Nat. Rev. Earth Environ..

[36] Saffer DM (2010). Hydrostratigraphy as a control on subduction zone mechanics through its effects on drainage: An example from the Nankai margin, SW Japan. Geofluids.

[37] Brown KM, Kopf A, Underwood MB, Weinberger JL (2003). Compositional and fluid pressure controls on the state of stress on the Nankai subduction thrust: A weak plate boundary. Earth Planet. Sci. Lett..

[38] Underwood MB, Dixon T, Moore JC (2007). Sediment inputs to subduction zones: Why lithostratigraphy and clay mineralogy matter. The Seismogenic Zone of Subduction Thrust Faults.

[39] Kitajima H, Saffer DM (2012). Elevated pore pressure and anomalously low stress in regions of low frequency earthquakes along the Nankai Trough subduction megathrust. Geophys. Res. Lett..

[40] Davis D, Suppe J, Dahlen FA (1983). Mechanics of fold-and-thrust belts and accretionary wedges. J. Geophys. Res..

[41] Koge H, Ashi J, Park J-O, Miyakawa A, Yabe S (2022). Simple topographic parameter reveals the along-trench distribution of frictional properties on shallow plate boundary fault. Earth Planets Space.

[42] Park J-O, Tsuru T, Kodaira S, Cummins PR, Kaneda Y (2002). Splay fault branching along the Nankai subduction zone. Science.

[43] Yu, F., Jamali Hondori, E. & Park, J.-O. Pore-fluid pressure estimation for the Nankai Trough plate-boundary fault: Implications for shallow very low frequency earthquakes. *Abstract of Japan Geoscience Union Meeting 2022*, SCG44-28 [Available at https://confit.atlas.jp/guide/event/jpgu2022/subject/SCG44-28/detail?lang=en] (2022).

