# Supplementary material for: Link between the Nankai underthrust turbidites and shallow slow earthquakes
Source: Sci Rep. 2023 Jul 10;13:10333. doi: 10.1038/s41598-023-37474-6 (PMC10333230; doi:10.1038/s41598-023-37474-6)
Supplement: Supplementary file 1 — Supplementary Figure S1. [file 41598_2023_37474_MOESM1_ESM.pdf]

Supplementary Information for

Link between the Nankai underthrust turbidites and shallow slow earthquakes

Jin-Oh Park* and Ehsan Jamali Hondori

*Corresponding author. E-mail: jopark@aori.u-tokyo.ac.jp

This Word file includes: Supplementary Figure S1

Supplementary Figure S1.
Time-migrated MCS profiles without interpretations perpendicular to the MCS line NT0501H. Vertical exaggeration about 5:1 at the seafloor. (**a**) MCS profile of line KR0114-5. An ODP site (1177) is projected. (**b**) MCS profile of line KR0114-3. (**c**) MCS profile of line KR0108-4. An IODP Site C0011 is projected. (**d**) MCS profile of line KR9806-10. Two ODP sites (808 and 1173) are projected.

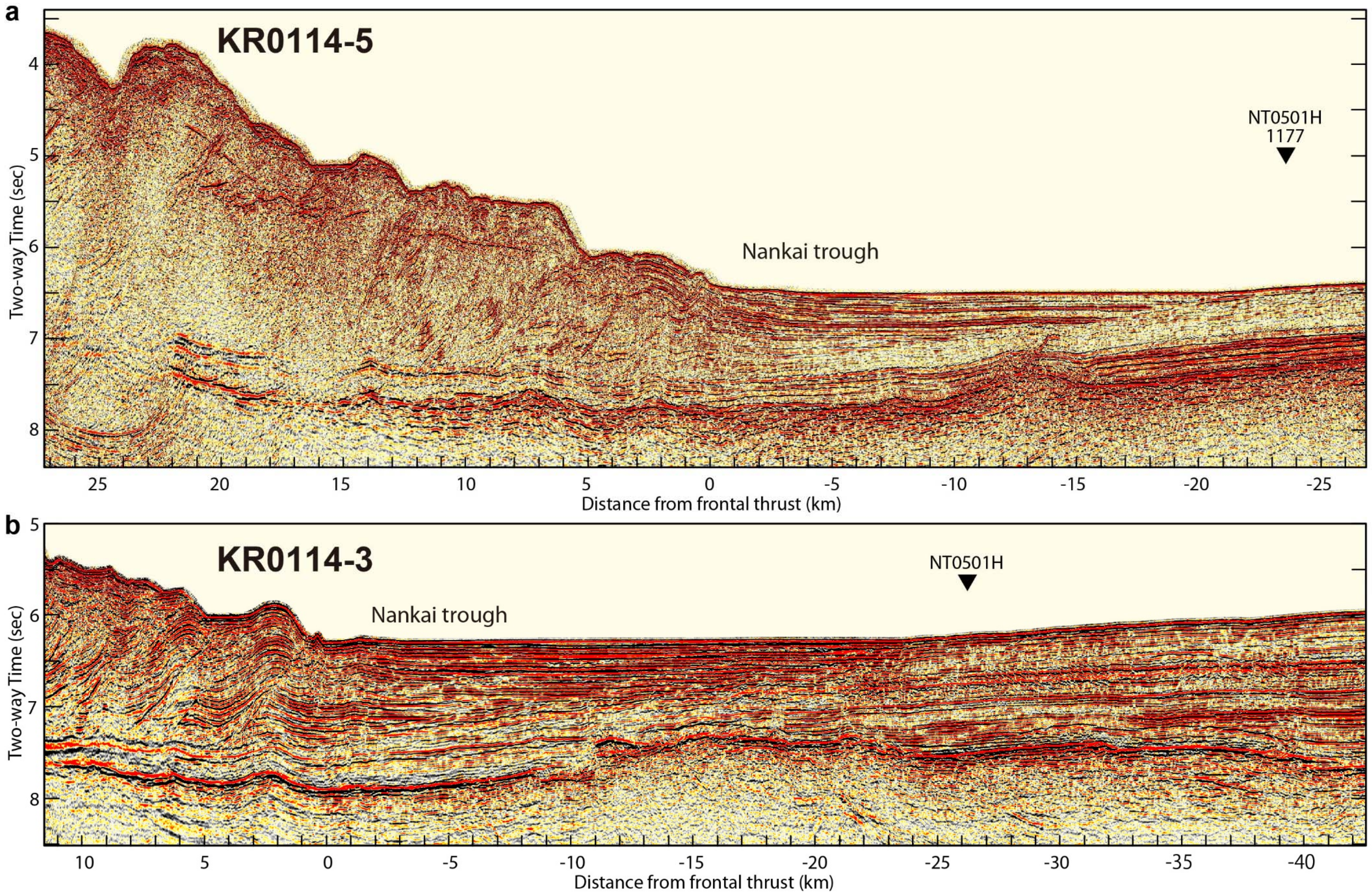
a
KR0114-5
NT0501H
1177
Nankai trough
Two-way Time (sec)
4
5
6
7
8
25
20
15
10
5
0
-5
-10
-15
-20
-25
Distance from frontal thrust (km)
b
KR0114-3
NT0501H
Nankai trough
Two-way Time (sec)
5
6
7
8
10
5
0
-5
-10
-15
-20
-25
-30
-35
-40
Distance from frontal thrust (km)

c

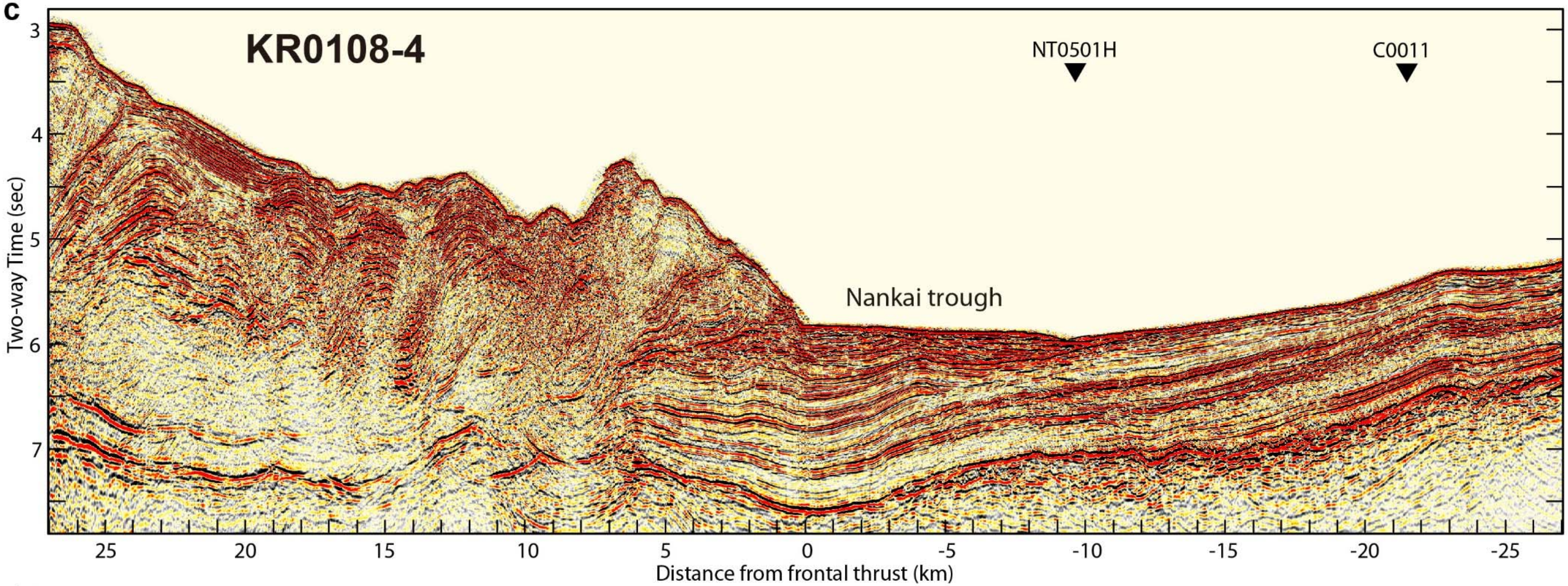
KR0108-4
NT0501H
C0011
Nankai trough
Two-way Time (sec)
3
4
5
6
7
25
20
15
10
5
0
-5
-10
-15
-20
-25
Distance from frontal thrust (km)

d

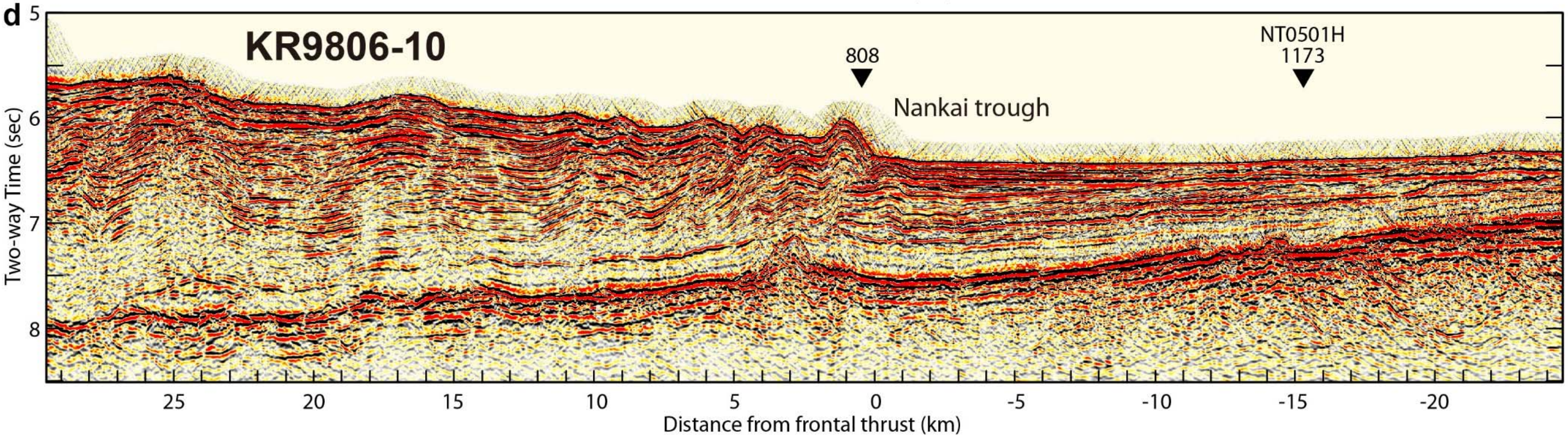
KR9806-10
808
NT0501H
1173
Nankai trough
Two-way Time (sec)
5
6
7
8
25
20
15
10
5
0
-5
-10
-15
-20
Distance from frontal thrust (km)
